# The Efficacy and Psychoneuroimmunology Mechanism of Camouflage Combined With Psychotherapy in Vitiligo Treatment

**DOI:** 10.3389/fmed.2022.818543

**Published:** 2022-05-27

**Authors:** Yuqian Chang, Shaolong Zhang, Weigang Zhang, Shuli Li, Chunying Li

**Affiliations:** Department of Dermatology, Xijing Hospital, Fourth Military Medical University, Xi'an, China

**Keywords:** vitiligo, camouflage, psychotherapy, quality of life, psychoneuroimmunology

## Abstract

**Background and Objectives:**

The efficacy of camouflage combined with psychotherapy and the underlying mechanisms are poorly understood in vitiligo management. This study aimed to investigate the joint efficacy and further explore psycho-neuro-endocrine-immune-skin interactions.

**Patients and Methods:**

In a prospective, non-randomized and concurrent controlled trial, patients were divided into two groups. Quality of life (QOL) was evaluated using the Chinese version of the Vitiligo Life Quality Index (VLQI-C). Serum levels of neuropeptides and cytokines were detected by enzyme-linked immunosorbent assay.

**Results:**

A total of 149 patients were included for final evaluation. After treatment for 4 weeks, total and subcategory quality of life scores in the intervention group were much lower than in the control group. Serum levels of neuropeptide-Y (NPY) and melanin-concentrating hormone (MCH) significantly decreased, and serum level of adrenocorticotropic hormone (ACTH) increased in both active and stable patients of the intervention group, but not in the control group. In addition, the serum levels of interferon-γ (IFN-γ), CXC chemokine ligand 10 (CXCL10), and interleukin-1β (IL-1β) decreased in both the active and stable patients of the intervention group and only in the active patients of the control group.

**Conclusions:**

The combination of camouflage and psychotherapy provided a clinically meaningful improvement in quality of life and ameliorated the outcome by likely modulating the psycho-neuro-endocrine-immuno-skin system during vitiligo management.

**Clinical Trial Registration:**

www.clinicaltrials.gov/ct2/show/NCT03540966, identifier: NCT03540966.

## Introduction

Vitiligo is a common autoimmune depigmentation of the skin with a preference for exposed sites (faces, hands, and feet) resulting in cosmetically disfiguring, psychologically disturbing and subsequently significant impairment of quality of life (QOL), posing a potent therapeutic demand for the treatment (1, 2). The melanocyte destruction in vitiligo is multifactorial, involving psychological, physical, and environmental stressors, immune dysfunction, abnormal metabolism, and genetic predisposition (1, 3). Notably, psychological stress is related to the onset, progression, relapse, and remission of vitiligo by altering the levels of catecholamines, neuropeptides, cortisol, and cytokines (4–7). Recently, an innovative study demonstrated that stress activates the release of norepinephrine from sympathetic nerves, which in turn causes melanocyte stem cells to shift into pigment-producing cells, prematurely eliminate the reservoir from hair follicles, and then induce hair graying (8). These findings prompt us to investigate the importance of maintaining a positive psychological state in the selection of management modalities.

Current vitiligo therapeutic approaches are dependent on regulating immune dysfunction with immunomodulators, reducing accumulative oxidative stress in lesions with antioxidants, and activating melanocyte regeneration by narrowband ultraviolet B irradiation (NB-UVB) or transplanting healthy melanocytes (9–11). However, rates of halting progression and regimentation of these conventional options are only approximately 50%–60%, and these options inevitably lead to the absence of compliance of patients in vitiligo practice (12, 13). Notably, a series of studies has demonstrated that both camouflage and psychotherapy can effectively alleviate the patients' burden by improving quality of life (14, 15), and camouflage application has been an essential part of vitiligo management in some countries (16–18). However, camouflage treatment has always been considered a dispensable cosmetic option for many patients in China based on our clinical observations because of lack of corresponding psychological construct and clear mechanism statement when patients are recommended to apply masking agents.

Considering the knowledge gap, we designed this prospective, non-randomized, concurrent controlled intervention trial to evaluate efficacy improvements of camouflage combined with psychotherapy, and to explore further the possible mechanism of psychoneuroimmunology regulation by measuring change in neuropeptide and cytokine levels.

## Patients and Methods

### Study Design, Patients, and Clinical Samples

**Figure 1:**
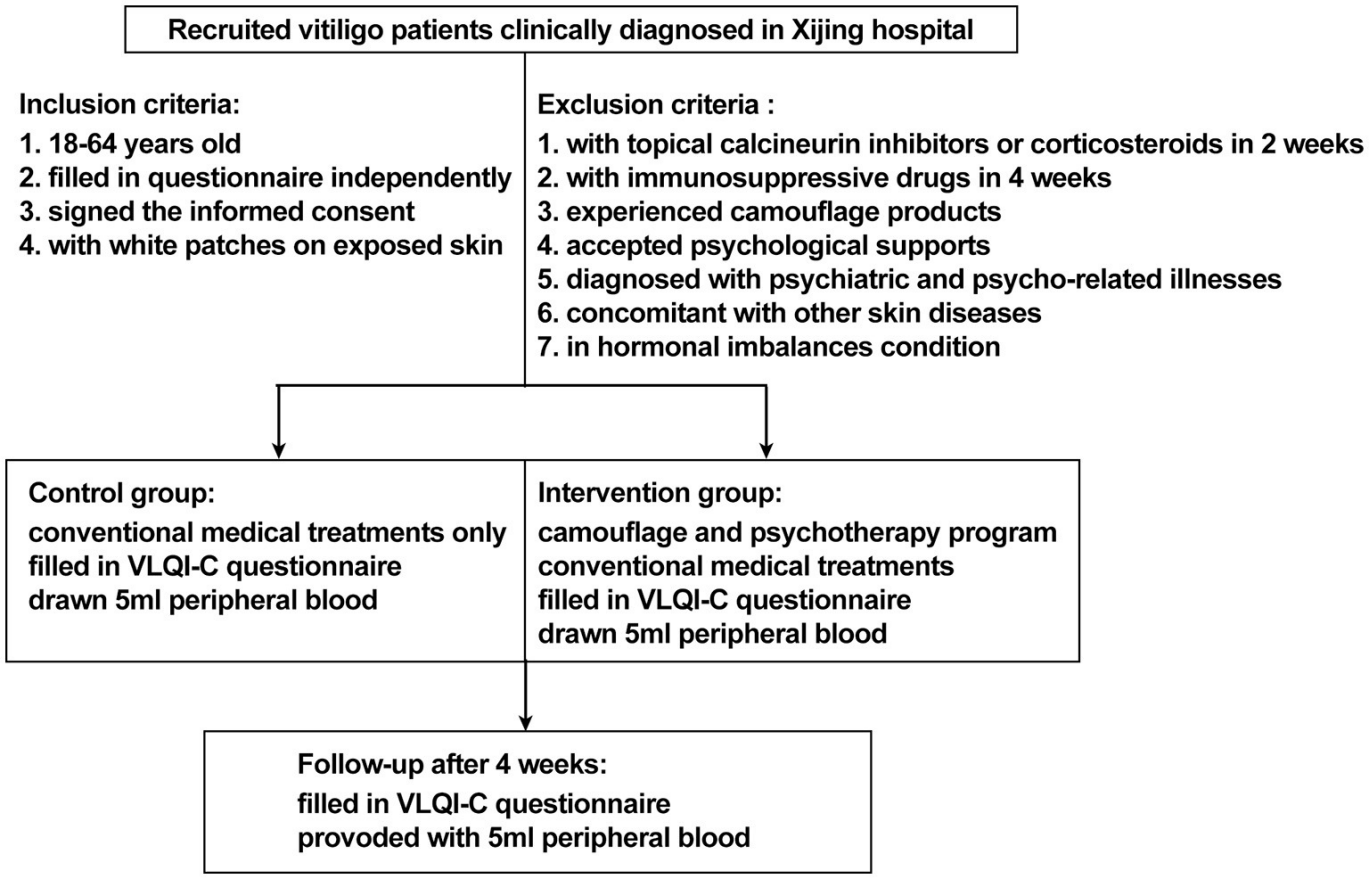
Overview of trial design.

**Figure 2:**
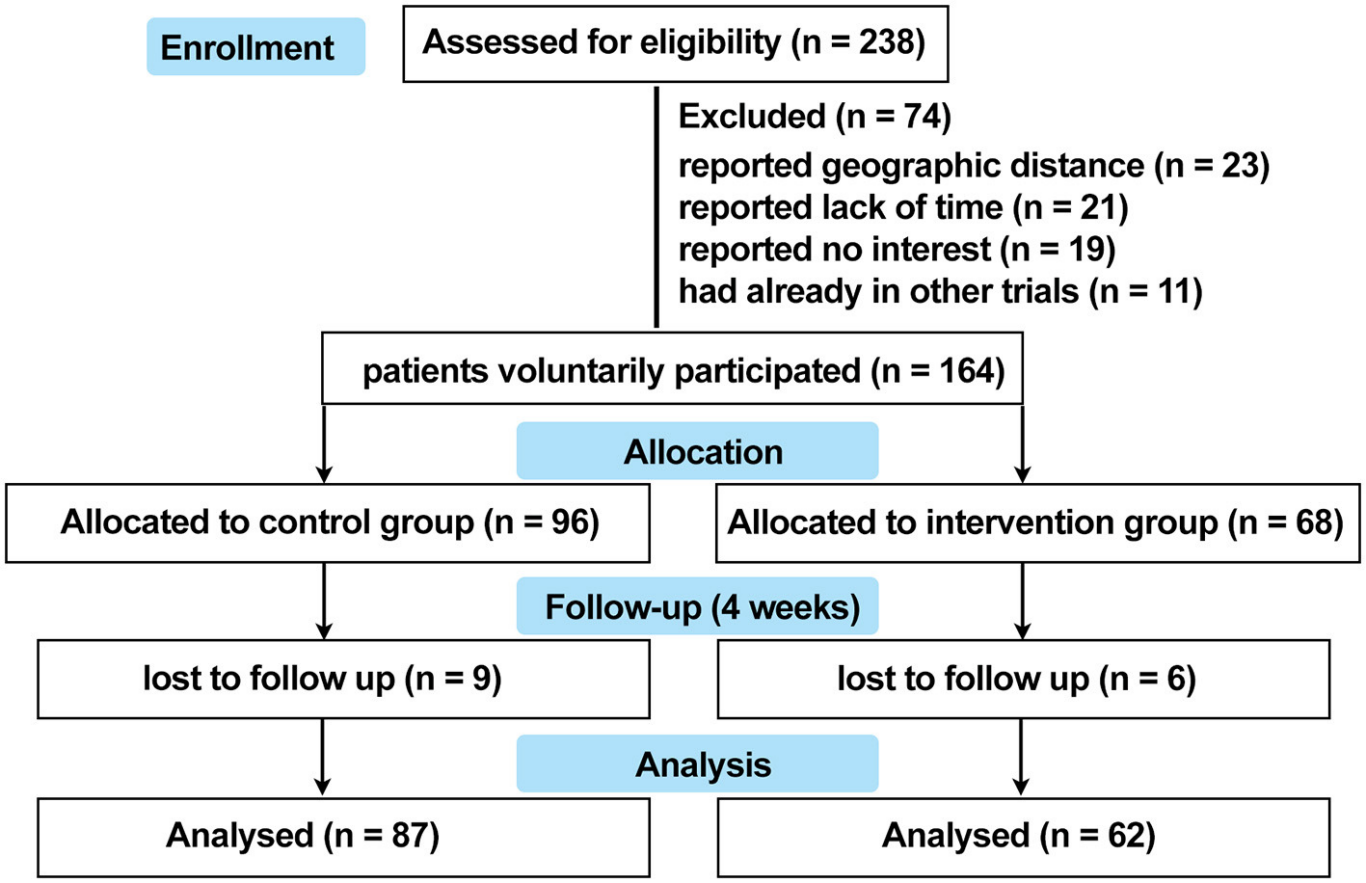
Flow chart to show cohort recruitment.

This study was designed as a prospective, non-randomized concurrent controlled trial that was reviewed and approved by the ethics review board of Xijing Hospital (approval code: KY20182005-1) and registered on ClinicalTrials.gov.cn (NCT03540966). A total of 238 patients with non-segmental vitiligo were recruited between May 2018 and December 2019 in Xijing Hospital. The key inclusion criteria were as follows: aged 18 to 60 years (read and filled out the questionnaire independently), diagnosed with vitiligo according to the consensus on the diagnosis and treatment of vitiligo in China (19), with vitiliginous patches on exposed sites (face, neck, hands, or feet where vitiligo can be recognized). The exclusion criteria were as follows: treatment with topical calcineurin inhibitors or topical corticosteroids within 2 weeks or immunosuppressive or immunomodulating medicines within 4 weeks, experienced any camouflage products to cover white patches or psychological support for vitiligo, diagnosed with psychiatric and psycho-related illnesses, concomitant with other autoimmune skin disorders such as atopic dermatitis, psoriasis, and alopecia areata, with QOL-threatening conditions such as cancer and chronic or autoimmune diseases, in hormonal imbalance contexts (women during pregnancy or menopause), or any other condition that the investigator deemed unsuitable for entering the study. The participants were assigned into two groups on a voluntary basis. Patients in the intervention group were treated with conventional medical treatments plus a camouflage and psychotherapy program, which was specifically designed for this trial, while patients in the control group received conventional medical treatment alone. All the patients were offered a follow-up at the dermatology clinic in Xijing Hospital 4 weeks later. For quantification of serum levels of neuropeptides and cytokines associated with vitiligo pathogenesis, 5-mL peripheral blood samples were collected from 10 patients in the active phase and from 20 patients in the stable phase of each group at both baseline and after 4 weeks of treatment. The patients were matched for age and gender. Protocols for human samples were performed according to the principles of the Declaration of Helsinki, and written informed consent was obtained from all the participants before initializing the treatment. The detailed trial design is shown in Figure 1, and the recruitment detail is shown in Figure 2.

### Camouflage and Psychotherapy Program and Procedures

The camouflage and psychotherapy program for this study was developed based on a literature review and experience summary by vitiligo experts (*n* = 7) in our dermatology department. All details were reviewed by the dermatologists and then validated in 9 patients with vitiligo. The program includes 4 dimensions: (1) one-to-one cognitive behavioral therapy for one session per week, (2) vitiligo popular science education and camouflage information provided as video tutorial, (3) camouflage skills training, and (4) answering of questions about vitiligo and camouflage *via* WeChat (communication software) during treatment. Through a series of procedures, the patients obtained psychological support, knowledge on vitiligo, and instructions for Capulin^TM^ (a commercial camouflage agent in China that mainly contains dihydroxyacetone, and the repigmentation mechanism involves polymerizing amino acids (histidine and tryptophan) of proteins and peptides in the stratum corneum, forming a special brown appearance within 8 h, characterized by good color-matching, 3–7-day longevity, simple application, and being waterproof and sweat-resistant). It should be noted that patients should use Capulin^TM^ 2 h before performing other topical management strategies to prevent interference with their respective effects. The camouflage and psychotherapy program and Capulin^TM^ are freely provided to all the participants in the intervention group.

### Conventional Medical Treatment for Patients

The choice of treatment depends on how much skin is involved and where and how quickly the disease is progressing. Generally, mid-potency topical corticosteroids are applied to the affected skin twice daily, and topical calcineurin inhibitors tacrolimus and pimecrolimus are used in place of depigmented macules involving the face, genitals, or any areas at high risk of skin atrophy. For patients with widespread or extensive lesions, NB-UVB phototherapy (with increase of 0.1 J/cm^2^ each time up to a maximum of 3 J/cm^2^ based on the initial dose 0.2 J/cm^2^; Phillips, Amsterdam, The Netherlands) was performed two to three times a week. For patients with minor lesions and those unable to go to an outpatient clinic room, smaller portable or handheld NB-UVB therapy devices are recommended for home use according to user guide.

### The VLQI-C Score Assessments

VLQI-C is a vitiligo-specific, cultural context-targeted QoL instrument for Chinese patients with vitiligo, and has been demonstrated to reflect the impact of vitiligo on patients' QoL with good internal consistency and temporal stability in another study (under review). The questionnaire contains 25 optional questions (Q1–Q25) that are further divided into 3 subscales named psychosomatic and social affects (PSAs, Q1–7, Q9, Q12, Q15–19, Q21–22, and Q24), self–perception (SP, Q8, Q10–11, and Q13–14), and disease management (DM, Q20, Q23), and 1 self-reported severity question (SRS: Q25) according to the results of exploratory factor analysis and contents of the items. Details of VLQI-C are provided in Supplementary Table 1. Each question is evaluated on a 4-point scale ranging from 1 (not at all) to 4 (very much), and total score ranges from 0 (best) to 100 (worst), where higher scores indicate worse quality of life. In this study, all the participants filled out the VLQI-C questionnaire by scanning the quick response code provided by their doctors and completed the questionnaire at both baseline and after 4 weeks treatment. Information on demographics, overall health, and vitiligo characteristics was also incorporated into the questionnaire.

### Detection of Neuropeptides and Cytokines by ELISA

Samples of 5-mL peripheral blood were collected with a serum-separating tube, left undisturbed to clot for 20–30 min at room temperature, and centrifuged at 3,000 rpm for 10 mins; the resulting supernatant was aspirated with a fresh polypropylene tube stored at −80°C for follow-up unified testing. The concentration of neuropeptide-Y (NPY), melanin-concentrating hormone (MCH), adrenocorticotropic hormone (ACTH), interferon-γ (IFN-γ), CXC chemokine ligand 10 (CXCL10), and interleukin-1β (IL-1β) was determined as previously described (20) using enzyme-linked immunosorbent assay (ELISA) kits (Labscience Biotechnology and Cusabio Biotechnology Co., Ltd, Wuhan, China) according to the manufacturer's instructions. Resultant absorbance was measured with a spectrophotometric microplate reader (Thermo Fisher Scientific, United States).

### Statistical Analysis

The IBM SPSS Statistics version 22.0 program was used for statistical analysis. Shapiro-Wilk test was conducted to test for normal distribution. For study population demographics, difference between the two groups was analyzed by Mann-Whitney U test. The association of each categorical variable with the study group was evaluated by Pearson's chi-squared test. Independent samples *t*-test was conducted to compare changes in VLQI-C scores between the two groups, and paired samples *t*-test was performed to compare changes in VLQI-C scores before and after treatment. Student's *t*-test is performed to compare the means between the two groups, and one-way ANOVA with Dunnett *post hoc* tests is conducted for groups of 3 or more. Two-tailed *P*-values were reported from all the *t*-tests.

## Results

### Characteristics of the Study Population

As a result, the data from a total of 149 patients with vitiligo were enrolled into the database, with 62 patients in the intervention group and 87 patients in the control group. The detailed overview of recruitment is shown in Figure 2. Full demographic information and baseline clinical characteristics of patients from both groups are summarized in Table 1. There was no statistically significant relationship between the two groups in terms of age, gender, education level, income level, marital status, exposited location, duration, lesion area, and phase (*P* >0.05).

**Table 1:** Demographics and baseline clinical characteristics of the patients.

**Variables**		**Groups**				***P***
		**Intervention**		**Control**		
		**Numbers/medium**	**Percentage/range**	**Numbers/medium**	**Percentage/range**	
Age		27	18–55	28	18–55	0.63
Gender	Male	27	43.5%	39	44.8%	0.88
	Female	35	56.5%	48	55.2%	
Education level	Primary school	3	4.8%	10	11.5%	0.53
	Middle school	13	21. %	19	21.8%	
	High school	38	61.3%	47	54.0%	
	College or higher	8	12.9%	11	12.6%	
Income level	<3,000	19	30.6%	24	27.6%	0.81
	3,000–5,000	19	30.6%	24	27.6%	
	5,000–10,000	16	25.8%	29	33.3%	
	≥10,000	8	12.9%	10	11.5%	
Marital status	Married	23	37.1%	41	47.1%	0.22
	Single	39	62.9%	46	52.9%	
Exposed site	Face/neck	27	43.5%	38	43.7%	0.89
	Hands/feet	24	38.7%	36	41.4%	
	Face/neck & hands/feet	11	17.7%	13	14.9%	
Duration (month)	<12	16	25.8%	16	18.4%	0.71
	12–60	20	32.3%	33	37.9%	
	60–120	18	29.0%	25	28.7%	
	≥120	8	12.9%	13	14.9%	
Depigmented macule area (face, neck, and hands) (BSA)	0–0.5	31	50.0%	36	41.4%	0.53
	0.5–1	25	40.3%	39	44.8%	
	≥1	6	9.7%	12	13.8%	
Phase	Stable	37	59.7%	57	65.5%	0.50
	Active	25	40.3%	30	34.5%	

### Camouflage and Psychotherapy Program Markedly Improved the QOL of Patients With Vitiligo

According to the VLQI-C evaluation, the baseline scores in the intervention group were slightly higher but without statistical significance than the corresponding scores in the control group (total: 65.27 ± 13.77 vs. 61.99 ± 12.2; PSA, 42.45 ± 11.1 vs. 40.13 ± 9.04; SP, 14.68 ± 3.16 vs. 14.03 ± 3.12; DM, 5.19 ± 1.13 vs. 4.91 ± 1.20; SRS, 2.95 ± 0.76 vs. 2.92 ± 0.85) (Table 2), suggesting that patients with lower level of QOL were more willing to accept the camouflage and psychotherapy program.

**Table 2:** Baseline levels of total and subscales scores of patients with vitiligo in the intervention group and the control group.

**Variables**	**Mean ± SD**	**95% CI**	***P***
Total score of IG	65.27 ± 13.77	−0.94–7.52	0.127
Total score of CG	61.99 ± 12.20		
PSA score of IG	42.45 ± 11.10	−0.94–5.59	0.162
PSA score of CG	40.13 ± 9.04		
SP score of IG	14.68 ± 3.16	−0.39–1.67	0.219
SP score of CG	14.03 ± 3.12		
DM score of IG	5.19 ± 1.13	−0.10–0.67	0.144
DM score of CG	4.91 ± 1.20		
SRS score of IG	2.95 ± 0.76	−0.24–0.30	0.813
SRS score of CG	2.92 ± 0.85		

*SD, standard deviation; CI, confidence interval; IG, intervention group; CG, control group; PSA, psychosomatic and social affect; SP, self perception; DM, disease management; SRS, self-reported severity*.

**Figure 3:**
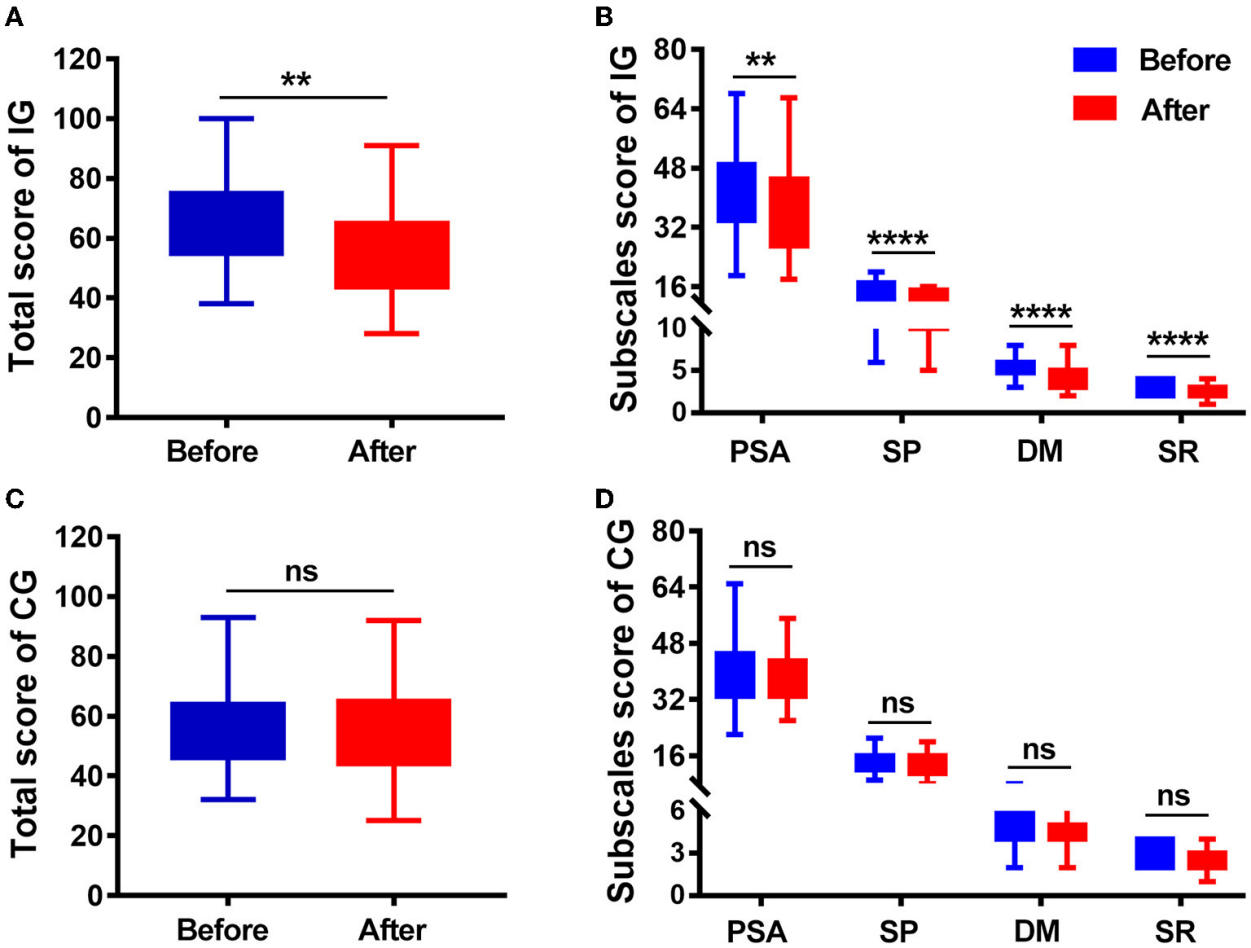
Quality of life (QoL) score at baseline (before) and 4 weeks later (after). **(A)** Total VLQI-C scores before and after camouflage and psychotherapy in the IG. **(B)** PSA, SP, DM, and SRS scores before and after camouflage and psychotherapy in the IG. **(C)** Total VLQI-C scores before and after 4 weeks in the CG. **(D)** PSA, SP, DM, and SRS scores before and after camouflage and psychotherapy in the CG. IG, intervention group; CG, control group; PSA, psychosomatic and social affect; SP, self perception; DM, disease management; SRS, self-reported severity. **P < 0.01, ****P < 0.0001, ns, nonsignificant.

The VLQI-C scores (total, 65.27 ± 13.77 vs. 55.97 ± 14.79; PSA, 42.45 ± 11.1 vs. 36.87 ± 11.98; SP, 14.68 ± 3.16 vs. 12.52 ± 2.78; DM, 5.19 ± 1.13 vs. 4.19 ± 1.34; SRS, 2.95 ±0.76 vs. 2.39 ±0.66) markedly decreased after 4 weeks of treatment in the intervention group, but there were no statistically significant differences (total, 61.99 ± 12.2 vs. 59.14 ± 10.35; PSA, 40.13 ± 9.04 vs. 38.51 ± 7.11; SP, 14.03 ± 3.12 vs. 13.23 ± 3.11; DM, 4.91 ± 1.2 vs. 4.66 ± 1.23; SRS, 2.92 ±0.85 vs. 2.75 ±0.81) in the control group (Table 3; Figure 3). These results indicated that the camouflage and psychotherapy program could improve the QOL of patients with vitiligo.

**Table 3:** Total and subscales scores evaluated before and after 4 weeks of treatment in the intervention group and the control group.

**Variables**	**Intervention group**			**Control group**		
	**Mean ± SD**	**95% CI**	***P***	**Mean ± SD**	**95% CI**	***P***
Total score of before	65.27 ± 13.77	3.68–14.94	0.002	61.99 ± 12.20	−0.33–6.04	0.079
Total score of after	55.97 ± 14.79			59.14 ± 10.35		
PSA score of before	42.45 ± 11.10	0.92–10.24	0.020	40.13 ± 9.04	−0.73–3.98	0.175
PSA score of after	36.87 ± 11.98			38.51 ± 7.11		
SP score of before	14.68 ± 3.16	1.17–3.15	<0.0001	14.03 ± 3.12	−0.07–1.68	0.071
SP score of after	12.52 ± 2.78			13.23 ± 3.11		
DM score of before	5.19 ± 1.13	0.58–1.42	<0.0001	4.91 ± 1.20	−0.07–0.58	0.128
DM score of after	4.19 ± 1.34			4.66 ± 1.23		
SRS score of before	2.95 ± 0.76	0.32–0.81	<0.0001	2.92 ± 0.85	−0.05–0.39	0.120
SRS score of after	2.39 ± 0.66			2.75 ± 0.81		

*SD, standard deviation; CI, confidence interval; PSA, psychosomatic and social affect; SP, self perception; DM, disease management; SRS, self-reported severity*.

### Camouflage and Psychotherapy Program Reduced NPY and MCH Levels and Upregulated the Level of ACTH in Patients With Vitiligo

**Figure 4:**
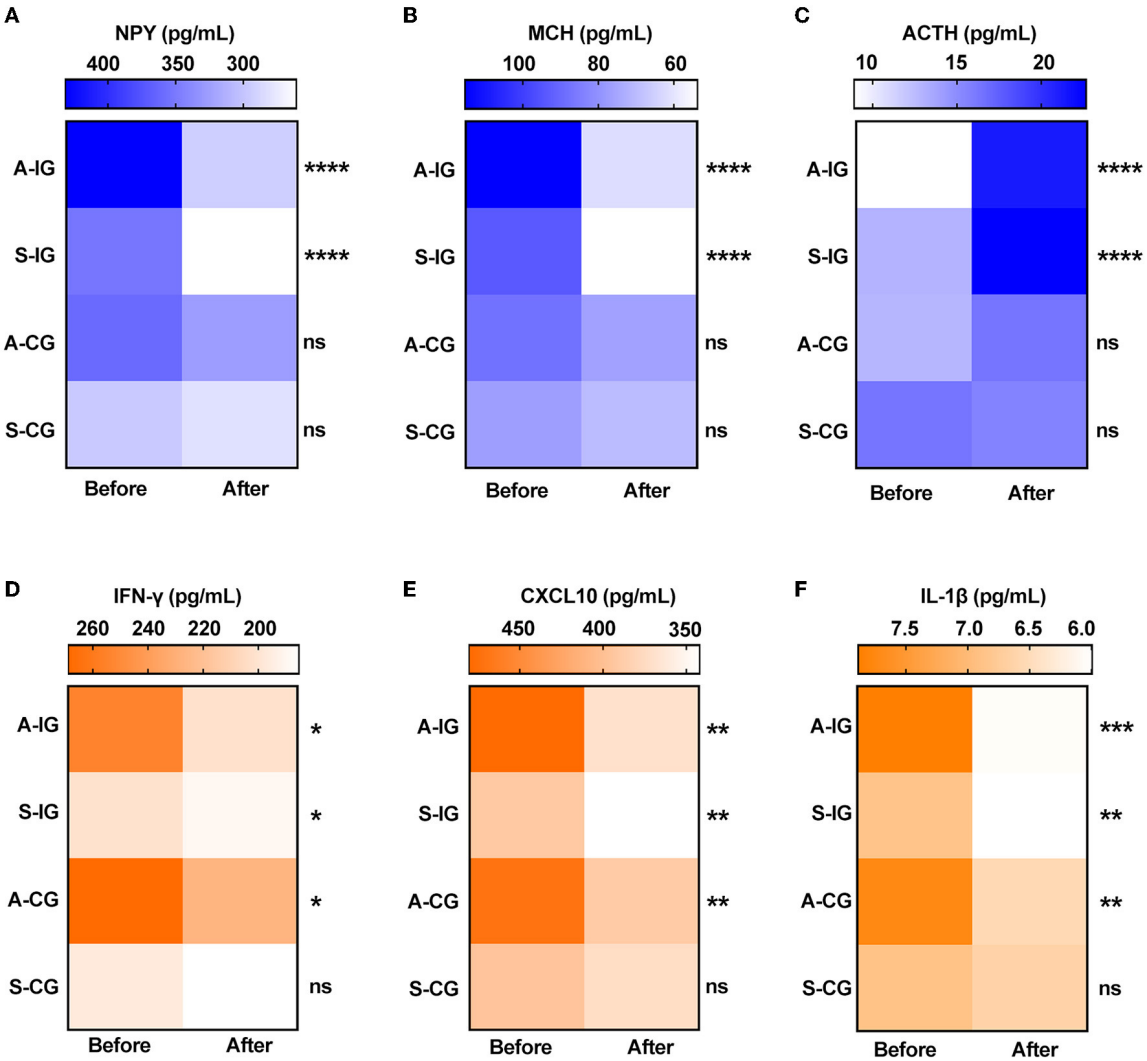
Serum levels of neuropeptides and cytokines before and after 4 weeks in the two groups. **(A)** NPY, **(B)** MCH, **(C)** ACTH, **(D)** IFN-γ, **(E)** CXCL10, and **(F)** IL-1β. A-IG, active phase of intervention group; S-IG, stable phase of intervention group; A-CG, active phase of control group; S-IG, stable phase of control group. *P < 0.05, **P < 0.01, ***P < 0.001, ****P < 0.0001, ns, nonsignificant.

Elevated level of NPY has been shown to be associated with vitiligo (21). We found that there was an obvious decrease in NPY serum level after 4 weeks of treatment in the intervention group; however, there was no significant change in the control group (Table 4; Figure 4A). MCH is the vital hypothalamic peptide that controls skin pigmentation (22). First, we identified a higher expression of MCH serum level in vitiligo cases as opposed to healthy controls (Supplementary Figure 1); subsequently, we observed a significant reduction of MCH level in the intervention group, whereas it was almost unchanged in the control group (Table 4; Figure 4B). Additionally, ACTH is involved in the response to biological stress, and its serum level is decreased in vitiligo sufferers compared with healthy controls (4). The results of this study demonstrated that ACTH serum level increased under the intervention of camouflage and psychotherapy, but that there was no obvious change in the control group (Table 4; Figure 4C). Taken together, camouflage and psychotherapy promote the restoration of neuropeptide levels in patients with vitiligo.

**Table 4:** Serum levels of NPY, MCH, ACTH, IFN-γ, CXCL10, IL-1β in patients with vitiligo of active (*n* = 10) and stable (*n* = 20) phases in both the intervention and control groups.

**Variables**	**Phase**	**Status**	**Intervention group**		**Control group**	
			**Mean ± SD**	***P***	**Mean ± SD**	***P***
NPY (pg/mL)	Active	Before	431.29 ± 46.93	<0.0001	420.36 ± 40.34	0.079
		After	293.35 ± 43.10		396.28 ± 51.40	
	Stable	Before	351.75 ± 49.25	<0.0001	336.63 ± 48.91	0.087
		After	260.80 ± 39.87		321.26 ± 61.57	
MCH (pg/mL)	Active	Before	115.26 ± 19.31	<0.0001	111.73 ± 20.25	0.050
		After	61.81 ± 11.61		100.24 ± 10.42	
	Stable	Before	93.74 ± 13.05	<0.0001	90.41 ± 12.19	0.059
		After	53.74 ± 9.92		83.47 ± 13.17	
ACTH (pg/mL)	Active	Before	8.89 ± 1.74	<0.0001	9.31 ± 3.25	0.064
		After	21.20 ± 3.72		11.61 ± 3.58	
	Stable	Before	12.97 ± 3.95	<0.0001	12.59 ± 2.43	0.075
		After	22.65 ± 5.01		14.42 ± 4.12	
IFN-γ (pg/mL)	Active	Before	254.14 ± 45.90	0.010	268.89 ± 48.66	0.013
		After	208.75 ± 27.62		226.41 ± 31.94	
	Stable	Before	213.26 ± 29.32	0.011	196.84 ± 34.56	0.131
		After	193.52 ± 24.00		185.27 ± 30.66	
CXCL10 (pg/mL)	Active	Before	480.33 ± 46.03	0.001	470.63 ± 40.76	0.001
		After	369.78 ± 34.75		389.77 ± 51.41	
	Stable	Before	392.37 ± 36.58	0.001	396.14 ± 45.05	0.082
		After	341.93 ± 44.52		373.04 ± 40.32	
IL-1β (pg/mL)	Active	Before	7.88 ± 0.64	<0.0001	7.74 ± 1.12	0.004
		After	6.05 ± 0.68		6.55 ± 0.60	
	Stable	Before	6.87 ± 0.90	0.004	6.88 ± 0.88	0.158
		After	5.99 ± 0.89		6.64 ± 0.59	

*NPY, neuropeptide-Y; MCH, melanin-concentrating hormone; ACTH adrenocorticotropic hormone; IFN-γ, interferon gamma; CXCL10, C-X-C motif chemokine ligand 10; IL-1β, interleukin 1; SD, standard deviation*.

### Camouflage and Psychotherapy Program Had a Synergistic Effect With Routine Care in Vitiligo Treatment by Down-Regulating the Expression of IFN-Γ, CXCL10, and IL-1β

IFN-γ, CXCL10, and IL-1β have emerged as dependable serum markers and play a critical role in T-cell response in vitiligo (23–25). Our results showed that the serum levels of IFN-γ, CXCL10, and IL-1β were significantly reduced in both the active and stable patients after camouflage and psychotherapy combined with conventional treatment for 4 weeks. However, only the active patients experienced the reduction in inflammation factors, not the stable patients within 4 weeks of convention treatment in the control group (Table 4; Figures 4D–F). Together, the results indicated that the intervention of camouflage and psychotherapy program could modulate inflammatory reactions in the short term.

## Discussion

This prospective, non-randomized, concurrent controlled study evaluated the efficacy of camouflage and psychotherapy intervention in patients with acrofacial vitiligo. Our study showed that patients with a relatively higher initial VLQI-C score preferred camouflage and psychotherapy and experienced significant reductions in VLQI-C scores within 4 weeks of treatment compared with those with routine care alone. Moreover, the significant changes in the NPY, MCH, ACTH, IFN-γ, CXCL10 and IL-1β serum levels induced by camouflage and psychotherapy in the patients with vitiligo indicated the mechanism of psycho-neuro-endocrine-immuno-skin interaction.

Lack of awareness and understanding of vitiligo in terms of etiology, pathogenesis, and treatment remains challenging for Asian patients (26, 27). Several studies have demonstrated that increasing access for psychotherapy including mental health counseling and cognitive behavioral therapy could markedly improve the quality of life of patients with vitiligo (28, 29). It was reported that vitiligo patients with facial involvement and higher Dermatology Life Quality Index (DLQI, a general questionnaire for all skin diseases) scores benefited from camouflage in a Belgian study; 40 vitiligo cases with camouflage showed a significant reduction in their DLQI scores compared to non-camouflaged users in an Egyptian study; camouflage lessons and self-camouflage for 4 weeks are effective for Japanese patients with vitiligo; psychological therapy for a period of 8 weeks has a positive effect on the progression of patients with vitiligo in a UK study (14). Recently, an observer-blinded self-controlled study from China demonstrated that camouflage can make significant improvement in QOL by assessing scores on DLQI and Vitiligo Impact Scale-22 (VIS-22, based on Indian patients) (30). It is worth noting that, here, we used a reliable and validated vitiligo-specific QoL instrument in the Chinese context. Our trial demonstrated improvement in QOL with the combination of camouflage and psychotherapy instead of a single therapeutic effect.

Melanocytes are derived from neural crest stem cells, so they have an embryologic link to the nervous system (31). Melanin synthesis is affected by several neurotransmitters and cytokines, and vitiliginous melanocytes are prone to neuro-endocrinal changes (4, 32). Psycho-neuro-endocrine-immuno-skin system is the study of the interactions among psychology, neural, endocrine, immune and skin processes (33, 34). Few studies have explored changes in neuropeptides and inflammatory cytokines after camouflage and/or psychotherapy interventions in vitiligo treatment. Psychological stress exposure resulting from vitiligo can lead to endocrine perturbation by altered neuropeptides and cytokines (33). These factors can, in turn, influence melanin synthesis by interacting with melanocytes and immune cells *via* their receptors (35–37).

NPY, a 36 amino acid neuromodulator secreted by neurons, is believed to be involved in control of vitiligo development (7, 21, 38, 39). Several studies have shown that the NPY levels of lesions and plasma in patients with vitiligo are significantly higher than in healthy volunteers (7, 21, 38). Furthermore, *NPY*−399T/C (rs16147) and +1128T/C (rs16139) polymorphisms are correlated with vitiligo susceptibility and increase NPY transcription activity (39, 40). In this study, we found that camouflage combined with psychotherapy could considerably decrease serum NPY level. MCH, a hypothalamic neuropeptide, controls skin pigmentation and is an antagonist of alpha-melanocyte–stimulating hormone-induced skin darkening (41, 42). Notably, in the serum of patients with vitiligo, autoantibodies against MCH receptor 1 (MCH-R1) were found to block the activity of MCHR1 and the binding of MCH with MCHR1 in a competitive manner (43). MCH mRNA expression in both lesion and non-lesion areas are upregulated in patients with vitiligo compared to healthy control, and MCH stimulation in human melanocytes reduces melanogenic actions (44, 45). However, the level of circulating MCH remains unknown in vitiligo. In this study, we primarily revealed a higher level of serum MCH in patients with vitiligo and discovered a reduced MCH level associated with camouflage and psychotherapy. ACTH, a prominent polypeptide hormone, stimulates the adrenal cortex, producing glucocorticoids to cope with stress (32). ACTH can cause dispersion of melanin granules (46), and prolonged administration of ACTH can induce skin hyperpigmentation (47, 48). There is a lower serum level of ACTH in either active or stable patients with vitiligo compared to healthy controls, which might be caused by the negative feedback regulation of higher serum levels of cortisol in active patients with vitiligo (4, 49). Our results demonstrated that camouflage and psychotherapy intervention markedly increased the level of ACTH.

Autoreactive cytotoxic CD8^+^ T cells specifically damage melanocytes and provoke the development and progression of vitiligo *via* presentation of IFN-γ and other cytotoxic effectors (50). Keratinocytes and fibroblasts stressed by IFN-γ can secrete chemokines CXCL9 and CXCL10, recruiting more CD8^+^ T cells to skin lesions (51–53). In addition, psychiatric factors may increase the release of IFN-γ in skin disorders (54). Hence, in this study, we observed the changes in IFN-γ and CXCL10 and found that the serum level of both decreased more drastically when combined with camouflage and psychotherapy. *IL1B*−511C/T SNP upregulates IL-1β transcript levels and results in increased risk for vitiligo. Moreover, IL-1β can induce biosynthesis and release of NPY; in turn, NPY can be involved in the humoral immune mechanism leading to a higher level of IL-1β in vitiligo (40, 55). In our previous study, we have demonstrated increased serum IL-1β levels in patients with vitiligo and correlated it with disease activity and severity, as well as a decrease after routine care (56). In this study, we found that IL-1β levels were reduced following camouflage combined with psychotherapy. These findings will help us understand the potential psycho-neuro-endocrine-immuno-skin system mechanism in management of vitiligo.

Some limitations were considered in the study. First, the number of patients was relatively small, as only 149 patients were willing to participate in the study. Second, the method for patient grouping was based on a voluntary basis instead of randomization of patients, because successful masking and allocation concealment are not possible because of the visibility of camouflage management. Future studies with larger samples might avoid selection bias through loss or withdrawal of randomized patients. Third, the study was limited to one region, and it would be difficult to conclude the results directly for people from the other regions and races. Last, long-term follow-up data are lacking, and we do not know if the benefit of psychotherapy in the intervention group was durable.

In conclusion, this study indicates that camouflage combined with psychotherapy may provide a valuable treatment modality that not only improves the quality of life but also enhances efficacy *via* psycho-neuro-endocrine-immuno-skin interactions in vitiligo management. Dermatologists should be aware of the importance of easing psychosocial burdens using cover products and psychological support during treatment of vitiligo.

## Data Availability Statement

The data presented in the current study are available from the corresponding author on reasonable request.

## Ethics Statement

The studies involving human participants were reviewed and approved by Ethics Review Board of Xijing Hospital (Approval Code: KY20182005-1). The patients/participants provided their written informed consent to participate in this study. Written informed consent was obtained from the individual(s) for the publication of any potentially identifiable images or data included in this article.

## Author Contributions

YC, SZ, WZ, SL, and CL were involved in the conception and design of the study. YC and SZ participated in the acquisition, analysis, and interpretation of data. YC drafted the manuscript. All authors critically revised the article for important intellectual content, contributed to the article, and approved the submitted version.

## Funding

The study was supported by National Natural Science Foundation of China (Grant Numbers: 12126606, 81803124, 81930087, and 81903207).

## Conflict of Interest

The authors declare that the research was conducted in the absence of any commercial or financial relationships that could be construed as a potential conflict of interest.

## Publisher's Note

All claims expressed in this article are solely those of the authors and do not necessarily represent those of their affiliated organizations, or those of the publisher, the editors and the reviewers. Any product that may be evaluated in this article, or claim that may be made by its manufacturer, is not guaranteed or endorsed by the publisher.
