# Supplementary material for: The Efficacy and Psychoneuroimmunology Mechanism of Camouflage Combined With Psychotherapy in Vitiligo Treatment
Source: Front Med (Lausanne). 2022 May 27;9:818543. doi: 10.3389/fmed.2022.818543 (PMC9198302; doi:10.3389/fmed.2022.818543)
Supplement: Supplementary file 1 [file Data_Sheet_1.doc]

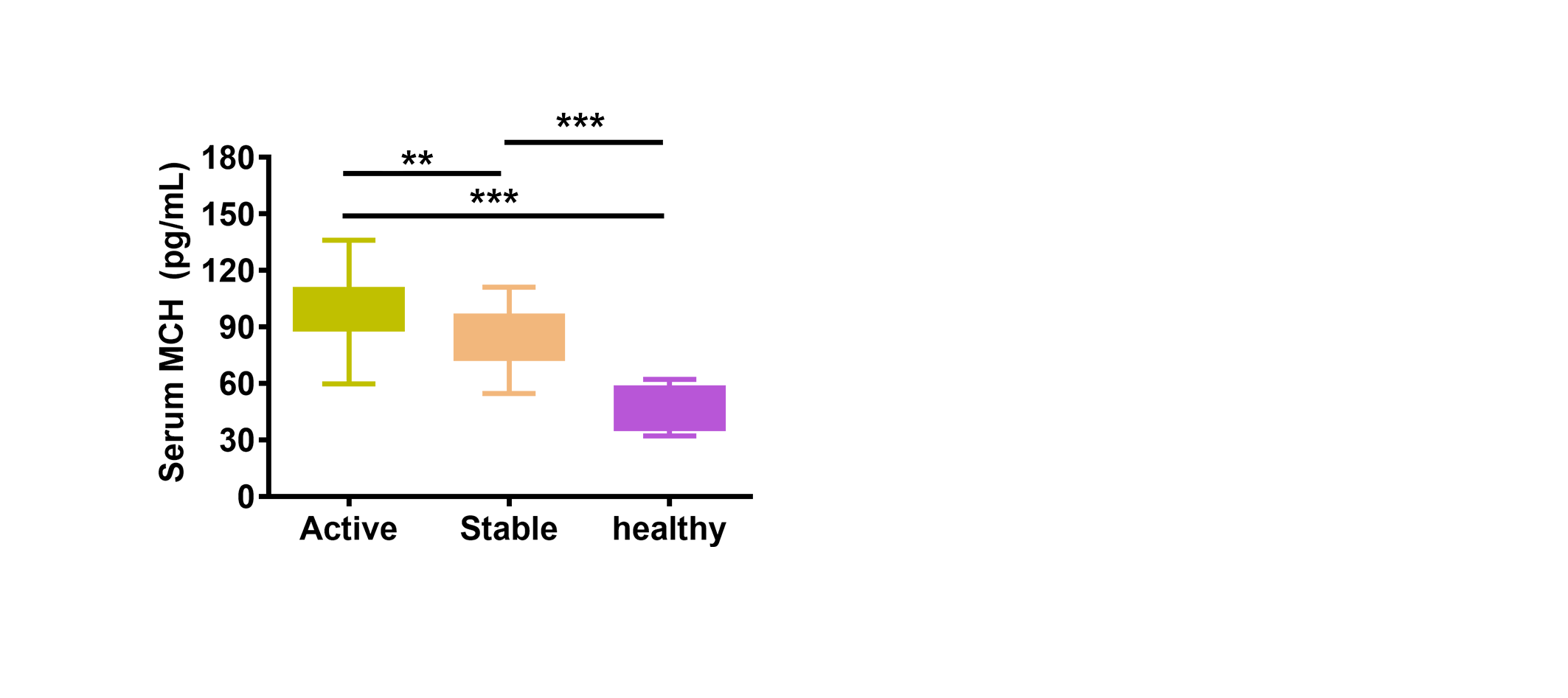
**Supplementary Material**

**Supplementary Figure** **1.** Serum levels of MCH of active and stable vitiligo patients and healthy controls. ***P*＜.01, ****P*＜.001.

**Supplementary Table 1** The detail of Vitiligo Life Quality Index of Chinese Version (VLQI-C)

This questionnaire is meant to measure the influence of vitiligo to your life. Please read every question carefully and answer them to the best of your understanding. It will take about 10 minutes. Scoring: 1. Not at all; 2. A little; 3. A lot; 4. Very much

| 25 Items | 1 | 2 | 3 | 4 |
| --- | --- | --- | --- | --- |
| 1. Have you felt depressed, frustrated or anxious (reduce work or activity, lethargy, insomnia and even want to end your life ), due to vitiligo? |  |  |  |  |
| 2. How much does vitiligo affect your daily life with family and friends? |  |  |  |  |
| 3. How much does vitiligo affect your appearance? |  |  |  |  |
| 4. Have you felt anxious the possibility of passing vitiligo on to your children? |  |  |  |  |
| 5. How much does vitiligo affect your sex life with your partner? |  |  |  |  |
| 6. Have you found it hard to bear the preconception from people around due to vitiligo? |  |  |  |  |
| 7. Has it cost you too much effort to treat vitiligo? |  |  |  |  |
| 8. Has it affected your mood when you see the white spots on skin? |  |  |  |  |
| 9. Have you felt uncomfortable when talking about vitiligo with people around? |  |  |  |  |
| 10. Have you found it difficult to maintain the treatment of vitiligo (spending too much time and money)? |  |  |  |  |
| 11. Has vitiligo affected your wardrobe choices (such as trying to choose clothing that covers a lot such as long pants, long sleeves, or wearing a hat or mask)? |  |  |  |  |
| 12. Have you felt like you can't accomplish anything because of vitiligo? |  |  |  |  |
| 13. Have you been worried about the appearance of the new white spots in other parts of the body? |  |  |  |  |
| 14. Have you worried about your vitiligo all the time? |  |  |  |  |
| 15. Have you felt it hard to make new friends or partners because of vitiligo? |  |  |  |  |
| 16. Has vitiligo brought too much stress or difficulties to your family or friends? |  |  |  |  |
| 17. Have you felt your vitiligo was particularly sensitive to the environment? |  |  |  |  |
| 18. Have you felt angry or drained without reason? |  |  |  |  |
| 19. Has vitiligo made you stay away from public place? |  |  |  |  |
| 20. Have you felt unsatisfactory with your current treatments? |  |  |  |  |
| 21. Have you felt isolated or ridiculed because of vitiligo? |  |  |  |  |
| 22. Have you refused to have physical contact with others because of vitiligo? |  |  |  |  |
| 23. Have you ever thought of abandoning the treatment of vitiligo? |  |  |  |  |
| 24. Have you spent more time to primp yourself in daily life? |  |  |  |  |
| 25. How do you feel about your current quality of life (vitiligo)? |  |  |  |  |
